# Measurement Equivalence between the Original and Estimated Mini-Mental State Examination in People with Dementia

**DOI:** 10.3390/ijerph18147616

**Published:** 2021-07-17

**Authors:** En-Chi Chiu, Tzu-Hua Chien, Ya-Chen Lee

**Affiliations:** 1 Department of Long-Term Care, National Taipei University of Nursing and Health Sciences, Taipei 112303, Taiwan; enchichiu@ntunhs.edu.tw; 2 Lian-Shun Home Care Nursing, Banqiao District, New Taipei City 220087, Taiwan; a0955667204@gmail.com; 3 Department of Occupational Therapy, College of Medical and Health Science, Asia University, Taichung 41354, Taiwan

**Keywords:** Mini-Mental State Examination, Cognitive Abilities Screening Instrument, cognition, dementia, measurement equivalence

## Abstract

Background: The Cognitive Abilities Screening Instrument (CASI) is increasingly used to assess general cognitive function in people with dementia. The Mini-Mental State Examination (MMSE) score can be converted from the CASI (i.e., the estimated MMSE). Recognizing that measurement equivalence is critical to meaningfully representing one with the other, we aimed to determine whether the estimated MMSE score obtained from the CASI was equivalent to the original MMSE in people with dementia. Methods: We obtained 110 data points for the MMSE and CASI scores in people with dementia. The intraclass correlation coefficient (ICC), Pearson’s *r*, percent of standard error of measurement (SEM%), paired *t*-test, and effect size (Cohen’s *d*) were used to investigate the equivalence. Results: To examine the equivalence between the original and estimated MMSE score, the ICC and Pearson’s *r* of the total score and six domains were 0.62–0.95 and 0.62–0.96, respectively. The SEM% of the total score and six domains were 0.6–8.9%. The paired *t*-test results showed a significant difference (*p* < 0.05) between the total score and the three domains. The Cohen’s *d* of the total score and six domains were 0.06–0.27. Conclusions: The estimated MMSE score was found to have moderate to excellent equivalence to the original MMSE score. The three domains (i.e., registration, attention and calculation, and visual-constructional ability) with moderate equivalence should be used cautiously to interchange with the original MMSE in people with dementia.

## 1. Introduction

As the world’s population ages, dementia is becoming a worldwide public health problem. The number of people with dementia is expected to increase to 66 million by 2030 and 115 million by 2050 [1]. Dementia is a neuropsychiatric disease characterized by general cognitive decline [1]. Cognitive dysfunctions in people with dementia include difficulties in memory, disturbances in language, and difficulties in attention and calculation, which result in impairments in activities of daily living [2,3]. As the disease progresses, different conditions of cognitive dysfunction affect the treatment and care provided. Therefore, to understand disease progression and whether the treatment and care meet the needs of people with dementia, it is important to choose a measuring tool that can assess general cognitive function.

The Mini-Mental State Examination (MMSE) and the Cognitive Abilities Screening Instrument (CASI) are widely used to assess cognitive impairment in people with dementia [4,5]. The MMSE contains six cognitive domains: orientation, registration, attention and calculation, recall, language, and visual-constructional ability [6]. The CASI was developed according to the MMSE, Modified Mini-Mental State test, and Hasegawa Dementia Screening Scale [7]. It is used to assess general cognitive function with nine domains: long-term memory, short-term memory, mental manipulation, orientation, attention, abstraction and judgment, language abilities, visual construction, and category fluency [7,8]. The MMSE and CASI are easy to administer and do not require specialized training or equipment. The administration time of the MMSE and CASI is about 5–10 min and 20–30 min, respectively [9,10]. The MMSE requires a shorter administration time; however, it is costly to use and does not assess long-term memory. Compared to the MMSE, the CASI includes four more domains (i.e., long-term memory, mental manipulation, abstraction and judgment, and category fluency). Evidence shows that the CASI can comprehensively assess general cognitive function [10]. In addition, the MMSE score can be converted from the CASI score (termed the estimated MMSE) [10]. The CASI appears to be an adequate tool for people with dementia in clinical and research settings.

Due to the above-mentioned advantages, the CASI is increasingly used in place of the MMSE when assessing people with dementia [7]. However, the measurement equivalence of the original MMSE and estimated MMSE has not yet been examined, limiting the utility of the CASI. Recognizing that measurement equivalence is critical to meaningfully represent one with the other, we aimed to determine whether the estimated MMSE score obtained from the CASI was equivalent to the original MMSE in people with dementia. We compared the total score and six domain scores from the original MMSE and estimated MMSE.

## 2. Materials and Methods

### 2.1. Participants

We recruited people undergoing a dementia daycare course between May 2017 and January 2018 in Northern Taiwan. The inclusion criteria were: (1) diagnosis of probable dementia according to the Diagnostic and Statistical Manual of Mental Disorders, fifth edition, (2) age ≥ 50 years, and (3) willingness to participate in the study. The exclusion criteria were diagnoses of intellectual disability and a history of severe brain injury. Moreover, the sample size was determined to be at least 50 participants to allow for more accurate interpretations of the associations between the original MMSE and estimated MMSE, as recommended in the literature [11,12].

Informed consent for participation was signed by the participants and their caregivers. This study was approved by the Institutional Review Board of Cardinal Tien Hospital. All methods of this study were performed per relevant regulations and guidelines.

### 2.2. Procedure

This study was part of the validation of the CASI study. People who met the inclusion were administered the MMSE and CASI twice, two weeks apart. The Clinical Dementia Rating (CDR) was administered to assess the severity of dementia in this study. The same examiner conducted all assessments. The assessments were conducted in a quiet place to avoid interference. Demographic data were collected from the medical records.

### 2.3. Measures

The MMSE measures general cognitive function. The maximum scores for the various domains range from 1 to 10 [6,13]. Together, the six domain scores total 30; a higher score indicates better general cognitive function [9]. The sensitivity and specificity of the MMSE have been examined in people with dementia [6].

The CASI was developed to assess general cognitive function. The maximum score for the various domains ranges from 8 to 18 [10]. The nine domain scores result in a total score of 100, with a higher score indicating superior general cognitive function [10]. Six domains of the CASI (i.e., short-term memory, mental manipulation, orientation, abstraction and judgment, language, and visual construction) were used to calculate the scores of the estimated MMSE [10]. The CASI has shown sufficient test-retest reliability in people with dementia [14].

The CDR measures cognitive and functional impairments in people with dementia. It contains six domains: orientation, memory, judgment and problem solving, community affairs, home and hobbies, and personal care [15]. The overall score estimates from the six domains to describe different severity levels of dementia: 0, healthy; 0.5, questionable dementia; 1, mild dementia; 2, moderate dementia; and 3, severe dementia [16]. The CDR has satisfactory reliability and validity in people with dementia [17].

### 2.4. Data Analysis

The intraclass correlation coefficient (ICC) with a 95% confidence interval was calculated to investigate the measurement equivalence between the original and estimated MMSE. An ICC value ≥0.90 indicates excellent equivalence, 0.75–0.89 indicates good equivalence, 0.50–0.74 indicates moderate equivalence, and <0.50 indicates poor equivalence [16]. Pearson’s *r* was calculated to examine the correlation between the original and estimated MMSE. Pearson’s *r* > 0.75 indicates good correlation, 0.50–0.75 indicates moderate correlation, and 0.25–0.50 indicates weak correlation [18]. The standard error of measurement (SEM) represents the measurement error between the original and estimated MMSE [19]. The SEM is computed as the standard deviation of all scores of assessments × √ (1-ICC). The percentage of SEM (SEM%) was calculated as (SEM/maximum value) × 100. The SEM% < 10% indicates a small measurement error between the original and estimated MMSE [19]. We applied a paired *t*-test (two-tailed, α = 0.05) and effect size (Cohen’s *d*) to investigate systematic bias between the original and estimated MMSE. A *d* value ≥ 0.80 displays large effect size, 0.50–0.79 displays moderate effect size, and 0.20–0.49 displays small effect size [20].

## 3. Results

A total of 57 participants participated in this study. Of these participants, 57 completed the first assessments and only 53 completed the second one, which yielded 110 data points obtained for the original MMSE and estimated MMSE. The mean age of the 57 participants was 83.1 years. The mean score of the CDR was 1.24, indicating that on average, our participants had mild-to-moderate dementia. The clinical characteristics of the participants are shown in Table 1.

**Figure 1:**
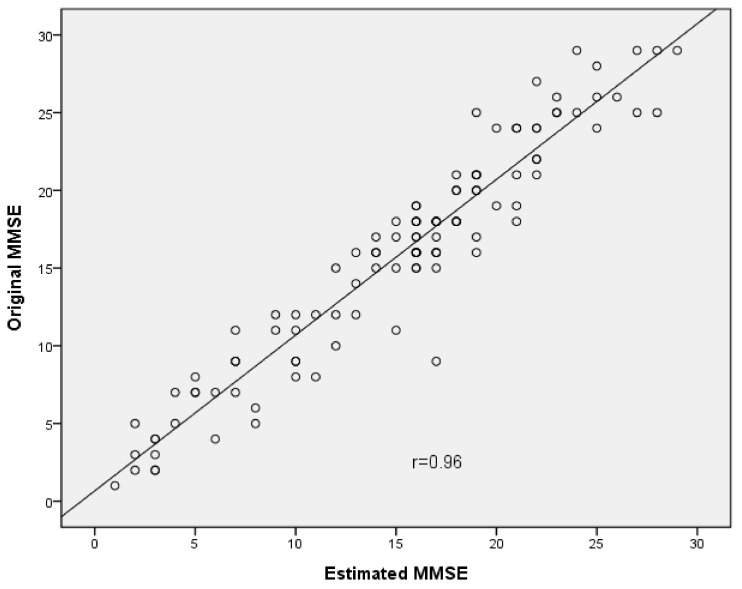
Scatter plot depicting correlation between total score of the Original MMSE and the Estimated MMSE.

The results of the equivalence between the original and estimated MMSE are presented in Table 2. The ICC values of the total score and three domains (i.e., orientation, recall, and language) in the original and estimated MMSE were 0.88–0.95. The ICC values of the three domains (registration, attention and calculation, and visual-constructional ability) were 0.62–0.64. The total score and the domains of orientation, recall, and language showed good correlations (*r* = 0.96, *p* < 0.001; *r* = 0.93, *p* < 0.001; *r* = 0.88, *p* < 0.001; *r* = 0.92, *p* < 0.001, respectively) (Figure 1). The three domains (i.e., registration, attention and calculation, and visual-constructional ability) showed moderate correlations (*r* = 0.63, *p* < 0.001; *r* = 0.62, *p* < 0.001; *r* = 0.66, *p* < 0.001, respectively). At the 95% confidence level, the SEM (SEM%) of the total score was 0.18 (0.06%). The SEM values and SEM% of all domains were 0.05–0.33 and 1.1–8.9%, respectively. For the paired *t*-test, there were significant differences in the total score and three domains (i.e., orientation, language, and visual-constructional ability) (*p* = 0.001–0.008) between the original and estimated MMSE. There were non-significant differences for the three domains (i.e., registration, attention and calculation, and recall) (*p* = 0.101–0.344) between the original and estimated MMSE. The *d* value of the visual-constructional ability domain was >0.20, and those of the total score and the other five domains were 0.06–0.14.

## 4. Discussion

This is the first study to investigate the measurement equivalence of the original and estimated MMSE in people with dementia. The results of ICC and Pearson’s *r* indicated that the total score and three domains (i.e., orientation, recall, and language) had good-to-excellent equivalence between the original and estimated MMSE. The other three domains demonstrated moderate equivalence between the original and estimated MMSE. The findings of this study imply that it may be appropriate to use the estimated MMSE to assess cognitive function in people with dementia.

Three possible reasons explain the moderate equivalence in the three domains (registration, attention and calculation, and visual-constructional ability). First, the ceiling effect was found in the registration domain of the original (81.8%) and estimated MMSE (76.4%). The ceiling effects could underestimate the correlations between the original and estimated registration domain [21]. To reduce ceiling effects, further studies may increase more difficult items in the MMSE and CASI [22]. Second, the attention and calculation domain of the original and estimated MMSE consists of different items. The attention and calculation domain of the original MMSE is scored using mathematical subtraction (i.e., serial subtraction of 7s), whereas this domain of the estimated MMSE is scored digitally backwards, not including mathematical subtraction. Moreover, mathematical subtraction in the CASI involves subtracting 3s serially, instead of subtracting 7s. These different items in the attention calculation domain between the original and estimated MMSE could influence their equivalence. Thus, we recommend that the mathematical subtraction domain in the CASI could be changed to a serial subtraction of 7s, and the attention and calculation domain of the estimated MMSE could include mathematical subtraction. Third, the scoring criteria are different in the original and estimated MMSE. For the visual-constructional ability domain, copying pentagons in the original MMSE scores a correct drawing (1 point) or incorrect drawing (0 points). Copying pentagons in the CASI scores with two criteria: drawing accuracy and crossover degree of two pentagons. Scores of 8–9 in copying pentagons in the CASI was converted to 1 point in the visual-constructional ability domain of the estimated MMSE. However, scores 8–9 are incorrect drawings, which are scored as 0 in the original MMSE. We re-analyzed and converted scores 8–9 and 10 of copying pentagons in the CASI as 0 and 1 point, respectively, for the visual-constructional ability domain of the estimated MMSE. The results demonstrated better equivalence in the original and estimated MMSE (ICC = 0.71 and *r* = 0.74). Therefore, the equivalence of both scales could be improved by converting the standard of the visual-constructional ability domain in the estimated MMSE. Copying pentagons with scores < 10 in the CASI is converted to 0 points, and 10 is converted to 1 point in the visual-constructional ability domain of the estimated MMSE.

The SEM% of the total score and domains were <10%, indicating a small measurement error between the original and estimated MMSE. The Cohen’s *d* values of the total score and domains were <0.20, except for the visual-constructional ability domain. The visual-constructional ability domain revealed statistically significant differences in the paired *t*-test and small effect size (*d* > 0.20), which demonstrates systematic bias between the original and estimated MMSE. This domain also showed relatively low ICC values and Pearson’s *r*, and thus the visual-constructional ability domain of the estimated MMSE may not be adequate to interchange with the original MMSE in people with dementia. Based on our overall findings, the estimated MMSE, except for the visual-constructional ability domain, showed sufficient equivalence with the original MMSE. In clinical implication, it is recommended to use the CASI to assess general cognitive conditions in people with dementia, with three advantages. First, the CASI includes more cognitive domains than MMSE. Second, the estimated MMSE scores can be obtained from the CASI. Third, the CASI is free to use.

This study has three limitations. First, we recruited participants from northern Taiwan, and the sample size was not large, which restricts the generalizability of the results in this study. Second, the participants included in this study were people with dementia; thus, the results of our study might not be generalizable to people with other types of cognitive dysfunction. Third, we used data where participants were administered the CASI and MMSE twice. Future studies are warranted to recruit a larger sample size, and for single administration of the assessments to each participant, to cross-validate our findings.

## 5. Conclusions

Our study demonstrated that the original and estimated MMSE had moderate to excellent measurement equivalence. These findings support the use of the estimated MMSE converted from the CASI for assessing general cognitive function in clinical and research settings. However, the three domains (i.e., registration, attention and calculation, and visual-constructional ability) showed moderate equivalence. Therefore, these three domains of the estimated MMSE should be implemented with caution when interchanging with the original MMSE in people with dementia.

## Figures and Tables

**Table 1:** Clinical characteristics of participants (n = 57).

Characteristic	
Age (years), mean (SD)	83.1 (7.9)
Gender, n (%)	
Male	28 (49.1)
Female	29 (50.9)
CDR, mean (SD)	1.24 (0.70)

SD, standard deviation; CDR, Clinical Dementia Rating.

**Table 2:** Equivalence between the original and estimated MMSE (n = 110).

Total Score and Domain Score	Original MMSE Mean ± SD	Estimated MMSE Mean ± SD	ICC (95% CI)	*r* (*p*-Value)	SEM (SEM%)	*t*-Test (*p*-Value)	Cohen’s *d*
Total score	15.88 ± 7.17	15.18 ± 6.85	0.95 (0.92, 0.97)	0.96 (<0.001)	0.18 (0.6%)	3.51 (0.001) *	0.10
Orientation	4.43 ± 2.98	4.15 ± 3.03	0.93 (0.90, 0.96)	0.93 (<0.001)	0.11 (1.1%)	2.70 (0.008) *	0.09
Registration	2.71 ± 0.67	2.61 ± 0.79	0.62 (0.49, 0.72)	0.63 (<0.001)	0.14 (4.6%)	1.65 (0.101)	0.14
Attention and calculation	2.17 ± 1.78	2.04 ± 1.67	0.62 (0.49, 0.72)	0.62 (<0.001)	0.33 (6.6%)	0.95 (0.344)	0.08
Recall	0.38 ± 0.89	0.33 ± 0.81	0.88 (0.82, 0.91)	0.88 (<0.001)	0.05 (1.7%)	1.35 (0.181)	0.06
Language	5.68 ± 2.28	5.43 ± 2.26	0.91 (0.87, 0.94)	0.92 (<0.001)	0.10 (1.1%)	2.92 (0.004) *	0.11
Visual-constructional ability	0.51 ± 0.50	0.64 ± 0.48	0.64 (0.50, 0.74)	0.66 (<0.001)	0.09 (8.9%)	−3.27 (0.001) *	0.27

SD, standard deviation; ICC, intraclass correlation coefficient; CI, confidence interval; SEM, standard error of measurement; MMSE, Mini-Mental State Examination. * Significant at *p* < 0.05.

## Data Availability

The data are not publicly available. The data presented in this study are available on request from the corresponding author.
